# Telemedicine in Disaster Medicine—Use of Paramedics as Tele-Consultants to Guide Personnel with Limited Medical Training at the Disaster Site

**DOI:** 10.3390/healthcare14203385

**Published:** 2026-10-11

**Authors:** Ulrike Walter, Lukas Weber, Michael Czaplik, Jan Larmann, Andreas Follmann, Anna Müller

**Affiliations:** 1 Faculty of Medicine, RWTH Aachen University, 52062 Aachen, Germany; 2 Docs in Clouds Telecare GmbH, 52074 Aachen, Germany

**Keywords:** telemedicine, disaster medicine, tele-Paramedic, tele-physician, mass casualty incident, tele-consultation, civil protection, simulation

## Abstract

**Background/Objectives:** Disaster medicine is frequently constrained by shortages of highly qualified medical personnel. Telemedicine may extend advanced expertise to less-qualified responders, but previous disaster telemedicine models have predominantly relied on physicians as remote consultants. This study compared patient care outcomes under telemedical support provided by experienced Paramedics with tele-physician support, conventional on-site physician care, and EMT-only care. **Methods:** A prospective, simulation-based, controlled quasi-experimental study was conducted during a 7.5-h mass casualty exercise in Hamburg, Germany. Seven treatment tents were assigned to four groups: on-site physician (CONT), EMT-only care (IV 1), tele-physician support (IV 2), and tele-Paramedic support (IV 3). Outcomes comprised time to first treatment order, indicated treatments addressed, analgesia management, and patient contact frequency by the most highly qualified team member. **Results:** A total of 151 simulated patients were included. Time to first treatment order did not differ significantly between groups (*p* = 0.09). The proportion of indicated treatments addressed differed significantly (*p* < 0.001), with rates of 56% in CONT, 8% in IV 1, 57% in IV 2, and 51% in IV 3. Analgesia was addressed in 68%, 7%, 59%, and 60% of patients with an indication, respectively (*p* < 0.001). Increasing pain intensity significantly increased the likelihood of analgesia in CONT, IV 2, and IV 3 but not in IV 1. Recorded patient contact frequencies were descriptively higher in the telemedicine groups, although contacts were operationally defined differently between telemedicine and non-telemedicine models. **Conclusions:** Tele-physician and tele-Paramedic support showed similar observed performance across selected outcomes to conventional physician-led care and substantially better rates of indicated treatments addressed and analgesia provision than EMT-only care. The findings indicate that experienced Paramedics may represent a valuable additional resource for telemedical disaster response. Larger studies are needed to confirm these results, define the appropriate scope of practice, and evaluate tiered consultation models in which tele-Paramedics provide first-line support and tele-physicians remain available for escalation of complex cases.

## 1. Introduction

Shortages of physicians and other highly qualified medical professionals like paramedics are already evident in routine patient care in most European countries [1]. These shortages may be exacerbated during disasters since, in Germany, civil protection units are staffed with volunteers [2]. Personnel with advanced medical qualifications are usually employed in different parts of the local healthcare system like emergency medical services (EMS) or hospitals. They may therefore be unable to participate in prehospital medical responses to a disaster [3].

Telemedicine has been implemented in EMS as one approach to addressing these shortages. In one approach, which has been in use since 2014, paramedics in ambulances can contact a tele-EMS physician at any given time when support is needed. This could be to receive a physician’s authorization to administer certain medications or to request telemedical support in life-threatening conditions. Telemedicine can also be used to bridge the time span until an EMS physician arrives on site [4].

In this regard, it is important to note that the term “paramedic” is often used colloquially to describe EMS personnel with heterogeneous levels of training and scope of practice. As these differences are relevant to the present study, a clear distinction will be made. For international comparability, qualification levels were classified according to the EMS education standards by the National Highway Traffic Safety Administration (NHTSA). German qualifications, though not completely equivalent, were assigned to the closest corresponding US provider levels based on training and scope of practice.

The paramedics receiving telemedical support in the EMS approach have undergone a three-year vocational training leading to the highest paramedic certification in Germany, comparable to a Paramedic defined by NHTSA [5]. They acquired extensive skills and legal rights to provide life-saving care, including invasive procedures and medications [6].

In disaster medicine, highly qualified personnel are a scarce and valuable resource. Providing telemedical consultation to providers who have completed only a three-month training program (competencies comparable to an Emergency Medical Technician (EMT)) may therefore help expand the workforce available to deliver medical care. These EMTs are primarily trained to provide basic medical services, including wound care, monitoring vital signs, and assisting more highly qualified healthcare professionals.

Previous studies have demonstrated that EMTs can successfully be guided via telemedicine by tele-physicians, thereby helping to mitigate physician shortages on site in disaster scenarios [7,8,9]. In the previous studies, the executing provider on site was referred to as a “tele-paramedic.” In the present study, this role is instead designated as telemedically connected EMT to more accurately reflect their level of training.

Whether the supporting telemedical professional must be a physician has not yet been investigated. However, non-physician telehealth models have been described in other acute and emergency care settings. A recent systematic review of telenursing in emergency and disaster settings identified applications in areas including triage, time-dependent emergencies, interhospital transport, and disaster response, with reported benefits regarding clinical outcomes, nursing autonomy, and resource utilization [10]. Paramedics may also be suitable tele-consultants for EMTs because of their broader clinical expertise and greater experience with complex pre-hospital decision-making. Here, consulting Paramedics with an expanded scope of practice acting as tele-consultants will be referred to as tele-Paramedics.

The objective of the present study was to compare patient-care outcomes under telemedical support provided by a tele-physician or tele-Paramedic with conventional on-site physician care and EMT-only care.

## 2. Materials and Methods

This prospective, simulation-based, controlled, quasi-experimental study with four parallel groups was conducted during a training exercise of civil protection units in Hamburg, Germany, in March 2024. The training exercise, organized by the German Federal Office of Civil Protection and Disaster Assistance, included a simulation of a mass casualty incident with simulated patients and dummies having injuries according to a predefined script. Each simulated patient first underwent triage and was subsequently allocated to a treatment tent by a dedicated transport coordination unit, primarily based on available treatment capacity. The study team had no influence on this allocation process. Allocation was therefore operational rather than randomized at the patient level. During the exercise, the team leader of one EMT-only tent requested that patients triaged as red not be assigned to that tent because no physician-level expertise was available there.

The simulation lasted 7.5 h. Study groups were randomly assigned across treatment tents.

The telemedicine set up in this study consisted of pairs comprising an on-site EMT and a remote consultant. Tele-consultants were either tele-physicians or tele-Paramedics. The tele-consultants had their workstations, equipped with a laptop with telemedicine app (TeleDoc Starter, version 3.11.1, Docs in Clouds TeleCare GmbH, Aachen, Germany), a second screen and a stereo headset, together in one conference room, enabling communication about organizational topics. However, discussing medical questions was prohibited so as not to distort the study results. The executing EMTs all had the same basic level of training. They were equipped with hardware and software enabling communication with their tele-consultant, including: a tablet with telemedicine app (TeleDoc Starter, Docs in Clouds TeleCare GmbH, Aachen, Germany), a mono headset, and a bodycam (Ulefone Night Vision Camera, Ulefone Technology Co., Ltd., Shenzhen, China). Audio and video were transmitted through the telemedicine app so each telemedicine duo could communicate orally, and the tele-consultants could see their executing EMTs’ surroundings. In addition, two digital medical devices were connected to the tablet via Bluetooth or cable to transmit vital signs via the telemedicine app. This included a multidevice with one-channel electrocardiogram and pulse oximetry (Viatom CheckmeTM Pro, Lepu Shenzen Viatom Technology Co., Ltd., Beijing, China) and an electronic stethoscope (Riester ri-sonic PCP-USB, Rudolf Riester GmbH, Jungingen, Germany).

**Figure 1:**
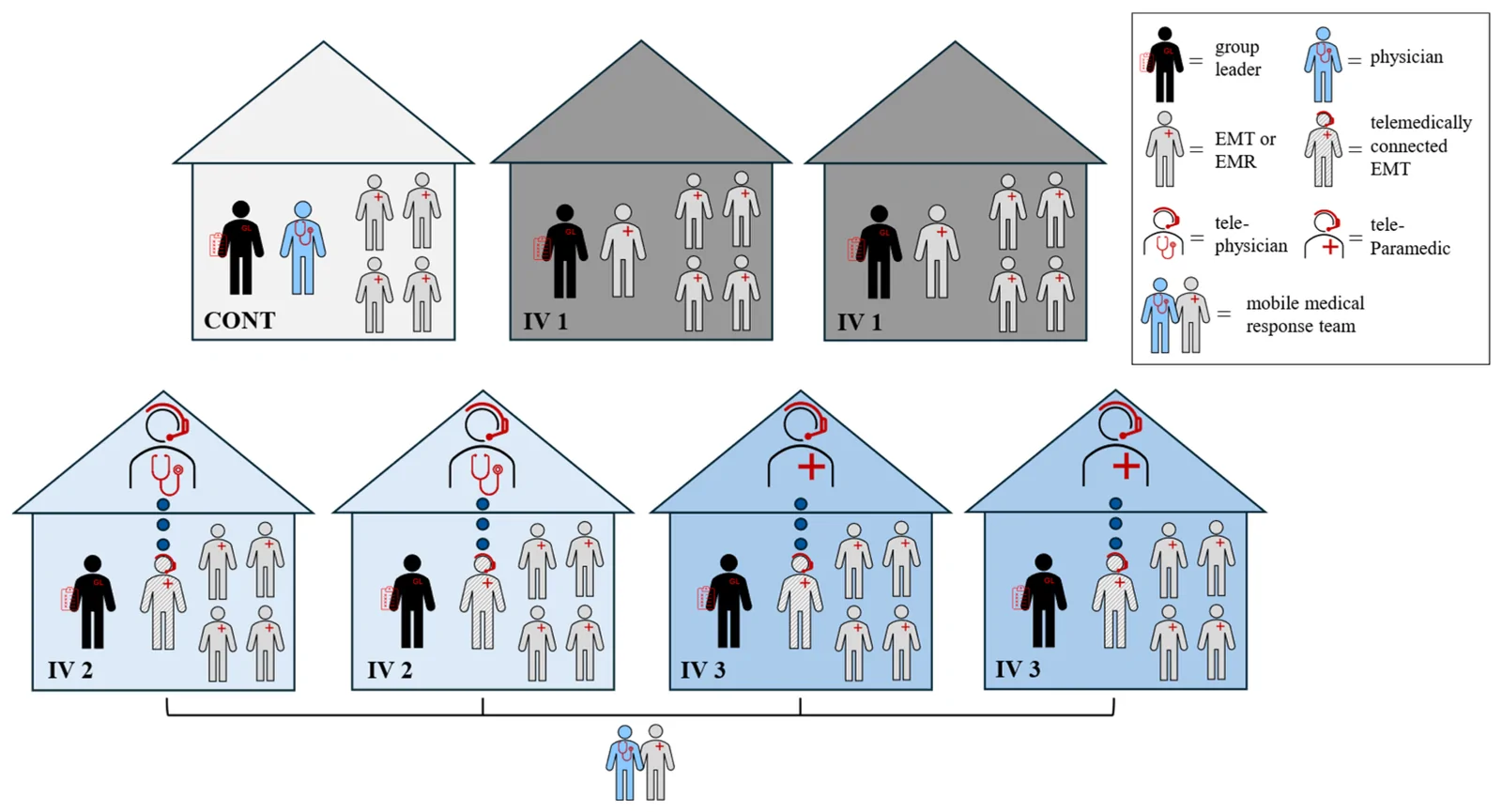
Composition of the physician (CONT, white), EMT-only (IV 1, grey), tele-physician (IV 2, light blue), and tele-Paramedic (IV 3, dark blue) study groups.

In this study, seven treatment tents were included (Figure 1). Each tent was designed to treat up to seven patients of different triage categories simultaneously and was staffed by six providers, including one team leader and four EMTs or Emergency Medical Responders (EMRs), with the proportion of EMTs and EMRs varying between tents. One tent was additionally staffed by an on-site physician, as conventionally intended (control group, CONT). Two tents were staffed by a fifth EMT, simulating a scenario in which no personnel with advanced medical expertise are available because of staff shortages (intervention group 1, IV 1). Furthermore, four tents were equipped with telemedicine. Of those, two tents were additionally staffed by an EMT supported by a tele-physician (intervention group 2, IV 2). The other two telemedicine tents were staffed by an EMT supported by a tele-Paramedic (intervention group 3, IV 3). Each telemedicine duo was instructed to maintain continuous consultation, allowing the tele-consultant to request ongoing updates on patients when considered necessary.

In addition, the four telemedicine tents were supported by a mobile medical response team comprising one mobile on-site emergency physician and one EMT serving as an assistant. The team functioned as an on-site escalation resource and provided direct clinical assessment and interventions whenever telemedical management was insufficient or infeasible. The team was intended for situations in which procedures could not be safely guided remotely, or for when the telemedicine provider was unavailable due to technical failure or simultaneous treatment of another patient. All study groups received a printed pocket card version of the German disaster medicine guidelines, which the tele-physicians and -Paramedics could use in digital form on their computers.

Each simulated patient or manikin received a unique four-digit identification number linked to their specific case, including the injury pattern, vital signs, and in some cases acting instructions. Injuries were created using professional moulage. Each patient also wore a neck card containing the case-specific clinical findings. Individual findings were concealed by removable stickers and could only be revealed after the corresponding examination had been conducted, thereby simulating clinical assessment and preventing providers from accessing information prematurely.

Patients were assigned predefined triage categories using the Primary Ranking for Initial Orientation in Rescue services (PRIOR) algorithm, a standardized prehospital structured primary-algorithm system used in German prehospital disaster response and designed for application by non-physician emergency personnel [11,12]. PRIOR follows an X-ABCDE-based assessment and incorporates select immediate life-saving interventions before assigning patients to red (immediate treatment is required because of an acute threat to life), yellow (urgent treatment is required without an immediate vital threat), green (treatment can be delayed), or black (patient is deceased) triage categories. Based on these predefined classifications, 22% of simulated patients were assigned to the red category, 34% to yellow, 43% to green, and 1% to black.

The study was embedded in a training exercise involving volunteers from civil protection units, organized by the federal state of Hamburg. A total of 46 medical personnel participated as EMTs, EMRs, an on-site physician, tele-physicians, or tele-Paramedics. Participant selection was not influenced by the study team. Six participants (5 EMTs and EMRs + 1 physician) were in the physician-on-site group; 12 participants (12 EMTs and EMRs) were in the EMT-only group; 14 participants (12 EMTs and EMRs + 2 tele-physicians) were in the tele-physician group; and another 14 participants (12 EMTs and EMRs + 2 tele-Paramedics) were in the tele-Paramedic group (Figure 1).

The study aimed to compare patient-care outcomes under telemedical support provided by either a tele-physician (IV 2) or tele-Paramedic (IV 3) with conventional on-site physician care (CONT) and EMT-only care (IV 1), the latter representing a disaster scenario in which no advanced medical expertise is available.

Firstly, the distribution of patients’ triage categories across the study groups was analyzed as a measure of baseline comparability between study groups.

The primary outcome was the time to first treatment order issued by the most highly qualified team member (CONT: physician; IV 1: most experienced EMT, IV 2: tele-physician, IV 3: tele-Paramedic). This outcome was chosen to specifically assess the influence of the qualification level of the most highly qualified team member on patient care.

Only treatment measures that would ordinarily require physician involvement were documented. Accordingly, only patients for whom at least one such treatment was issued by the most highly qualified team member were included in this analysis. In the telemedicine groups, telemedicine availability was assessed for each patient during the interval between arrival in the treatment tent and the first treatment order. The start and end of periods of telemedicine unavailability were identified from the tele-consultants’ screen recordings, which provided exact timestamps of system availability and unavailability. For each patient, all periods of telemedicine unavailability occurring within this interval were summed and subtracted from the elapsed time between arrival in the treatment tent and the first treatment order. The uncorrected times to first treatment order were reported as a sensitivity analysis.

In addition, the following secondary outcomes were analyzed and compared across all four study groups:

- Association between time to first treatment order by the highest qualified professional and patients’ triage category.
- The proportion of indicated treatments addressed was evaluated for thermal and traumatic injuries, focusing on the most frequently indicated interventions, including analgesia. For this analysis, a treatment was considered addressed if it was either ordered or physically performed, accounting for variation between teams in the enactment of treatment orders during the simulation.
- Quality of analgesia management: This outcome comprised the provision of indicated analgesia in patients with pain, the influence of pain severity (Numeric Rating Scale, NRS) on analgesia administration, the time to analgesia order including its association with patient triage category, and, in the telemedicine groups, the origin of the analgesia order (tele-consultant vs. mobile medical response team). As with the analysis of indicated treatments addressed, analgesia was considered provided if it was either explicitly ordered or physically administered.
- Patient contact frequency was analyzed. Patient contact was defined in the non-telemedicine groups (CONT + IV 1) as each instance in which the highest qualified professional moved from one patient’s bedside to another’s. In the telemedicine groups (IV 2 + 3), contact was counted each time the tele-consultant switched the conversation to a different patient. Contact frequency was expressed as the number of patient contacts per hour.

Study condition was assigned at the treatment-tent level, resulting in clustering of patients within seven treatment tents. Because only seven clusters were available, including a single control tent, cluster-adjusted models were unlikely to provide reliable estimates and were therefore not used for primary inference. Inferential analyses were consequently conducted at the patient level and are interpreted cautiously in view of the potential intracluster correlation.

All statistical analyses were performed using IBM SPSS Statistics for Windows, version 31.0.0.0 (IBM Corporation, Armonk, NY, USA). Data distribution was initially assessed using the Shapiro–Wilk test for normality. As most continuous variables were not normally distributed, between-group comparisons were performed using the Kruskal–Wallis test. Following a significant omnibus test, post hoc pairwise comparisons were conducted using Dunn’s test with Bonferroni correction for multiple testing. Categorical variables were analyzed using Pearson’s chi-squared test. Following a significant omnibus chi-squared test, pairwise comparisons were performed using Pearson’s chi-squared tests with Bonferroni correction. Fisher’s exact test was used when at least one expected cell frequency was below 5. For the proportions of indicated treatments addressed and patients with analgesia addressed, group-specific 95% confidence intervals were calculated using the Wilson score method. Absolute between-group differences in proportions were expressed in percentage points with 95% confidence intervals calculated using the Newcombe method. Binary logistic regression was used to assess the association between pain intensity (NRS score) and analgesia administration. Odds ratios (ORs) with 95% confidence intervals (CIs) were calculated. Linearity of the association between NRS and the log odds of analgesia administration was assessed using the Box–Tidwell approach by including an interaction term between NRS and its natural logarithm. Although the NRS is formally an ordinal scale, it was treated as a continuous predictor to assess the trend in analgesia administration across increasing pain levels.

All primary and secondary outcome parameters were prespecified in the study protocol. Bonferroni correction was applied within families of post hoc pairwise comparisons, whereas no global multiplicity adjustment was applied across the prespecified secondary outcome measures; these findings were therefore interpreted cautiously.

Statistical significance was defined as a two-sided *p*-value < 0.05. For all statistically significant results, effect sizes were calculated and interpreted according to the recommendations proposed by Cohen [13].

Data collection was performed through several means. In all seven treatment tents, 360° cameras were installed on the ceiling. The recorded videos were later used to identify the procedures each simulated patient received, the time when they were first ordered and when they were completed, and whether they were ordered by the highest skilled staff member inside the treatment tent.

Observers were placed in front of the tent doors to list the ID numbers, and time of entry and departure of the simulated patients. This allowed each patient and the corresponding case information to be matched to the 360° video recordings.

In the telemedicine groups (IV 2 + 3), the screens of the tele-consultants were recorded including their audio tracks. Afterwards, these recordings were reviewed to extract the time at which each procedure was first ordered by the tele-consultant, since this information could not be drawn from the 360° videos. The recordings also allowed the start and end of each period of telemedicine unavailability to be identified using the corresponding timestamps.

In the non-telemedicine tents (CONT and IV 1), observers shadowed the physician and the two most highly qualified EMTs. They listed the procedures performed, the time at which each procedure was first ordered and subsequently completed, and the number of times the observed practitioner changed bedside to assess a different patient.

All means of data collection were used to create one complete data set.

## 3. Results

A total of 151 patients were brought into the scenario progressively. Because the availability of treatment data varied by case, patients were included only in analyses for which the relevant data were available. The dataset was not adjusted in hindsight.

### 3.1. Distribution of Triage Categories

**Figure 2:**
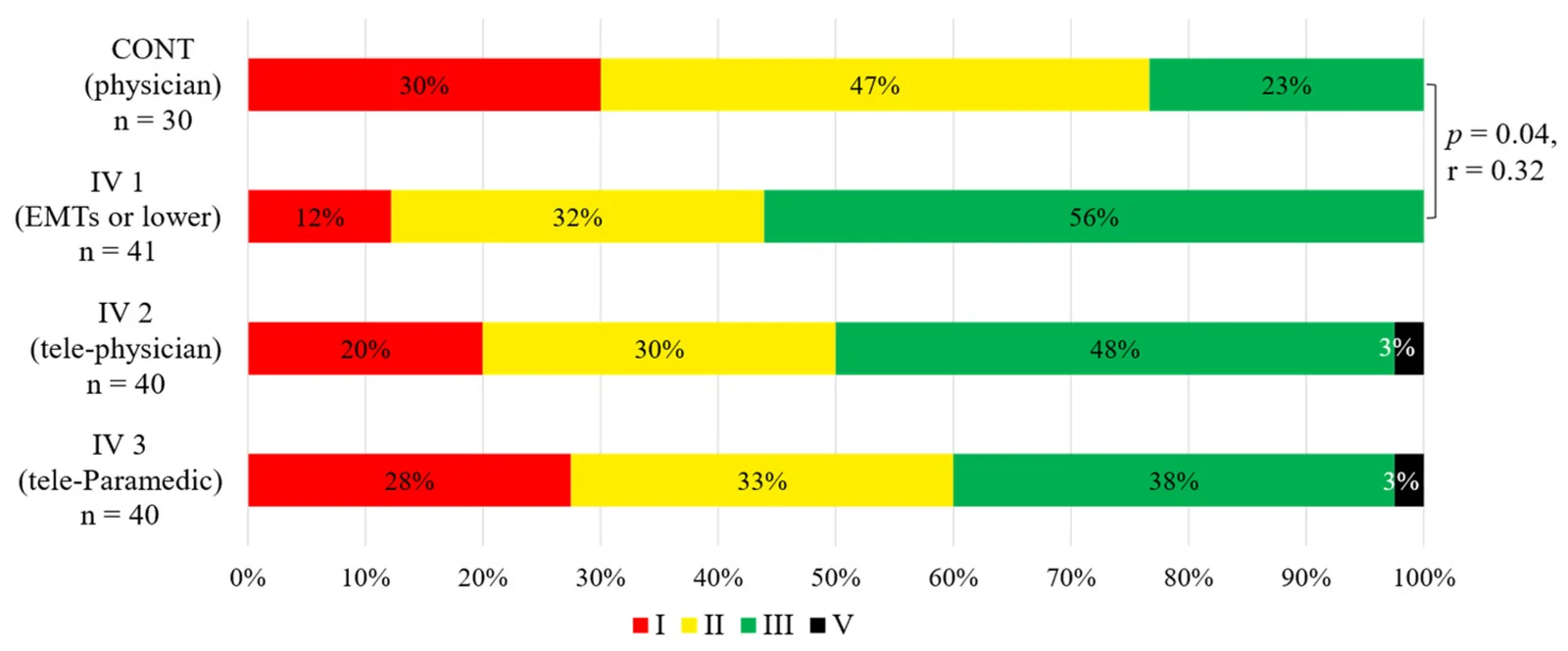
Triage categories of patients in each study group. The overall distribution differed significantly between study groups (Kruskal–Wallis, *p* = 0.040). The only significant post hoc comparison was between CONT and IV 1 (Dunn’s test with Bonferroni correction, adjusted *p* = 0.041, r = 0.32).

The distribution of triage categories varied across the four study groups (Figure 2). A Kruskal–Wallis test indicated a statistically significant difference in the distribution of triage categories across study groups (*p* = 0.04). Post hoc pairwise comparisons using Dunn’s test with Bonferroni correction identified a single significant difference between CONT and IV 1 (*p* = 0.04), with a small-to-moderate effect size (r = 0.32).

### 3.2. Time to First Treatment Order by Most Highly Qualified Team Member

**Figure 3:**
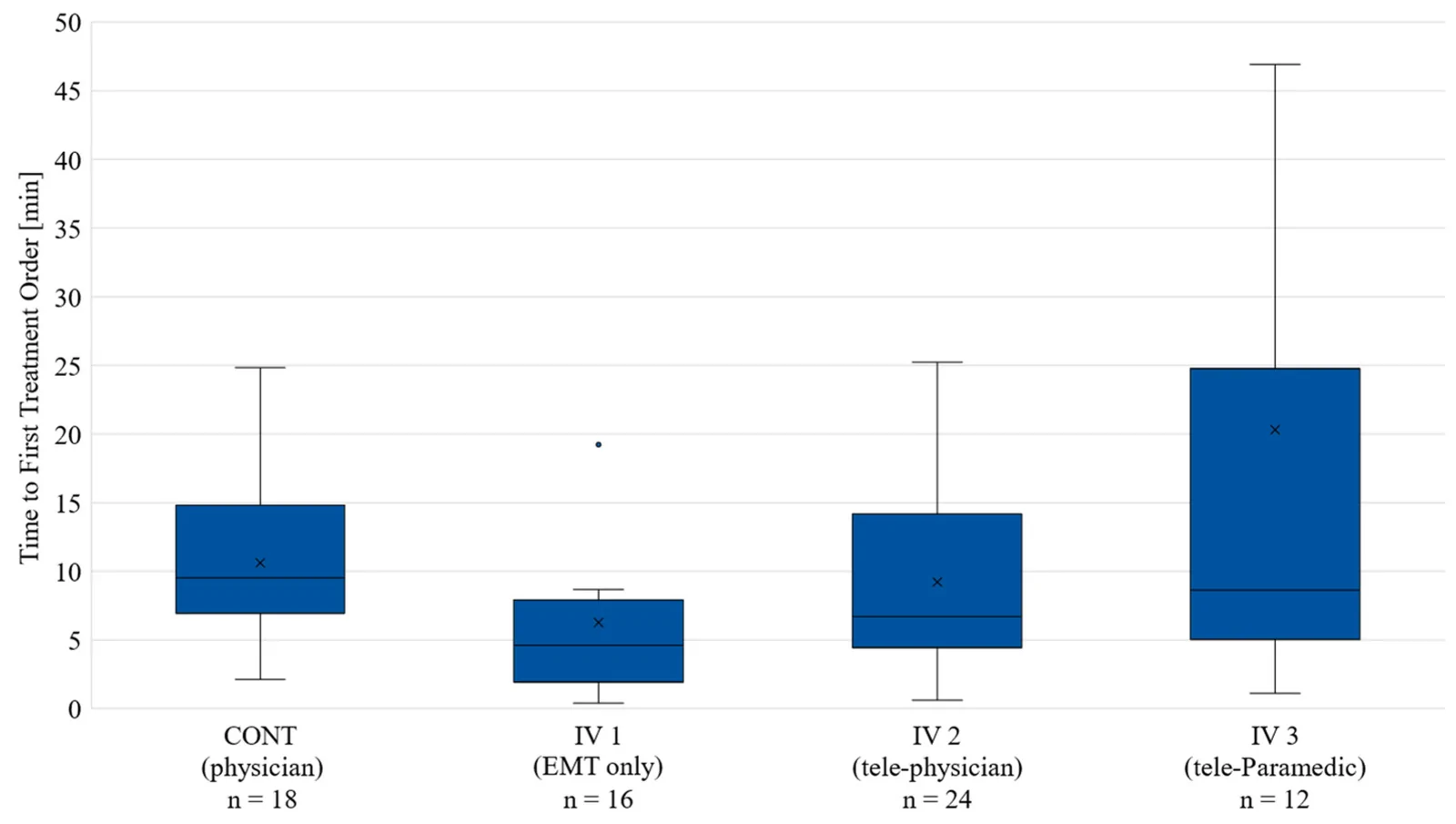
Time to first treatment order by most highly qualified team member. Boxes represent the interquartile range, horizontal lines indicate the median, and crosses indicate the arithmetic mean. Whiskers extend to the most extreme values within 1.5 times the interquartile range. Points beyond the whiskers represent statistical outliers.

The median time to first treatment order, corrected for telemedicine unavailability, by the most highly qualified team member was shortest in IV 1 and longest in CONT (Figure 3). In IV 1, the median time was 4.6 min (IQR 1.9–7.9), followed by IV 2 (6.7 min, IQR 4.45–14.18), IV 3 (8.6 min, IQR 5.0–24.8), and CONT (9.5 min, IQR 6.9–14.8). In IV 3, one outlier of 103.4 min was omitted from the figure for scaling purposes. Differences between groups did not reach statistical significance (Kruskal–Wallis; *p* = 0.09).

As a sensitivity analysis, time to first treatment order was additionally analyzed without correcting for periods of telemedicine unavailability. Because telemedicine downtime was not applicable to CONT or IV 1, values in these groups remained unchanged. In IV 2, the median uncorrected time increased from 6.7 min (IQR 4.5–14.2) to 10.1 min (IQR 5.0–22.0), while in IV 3, it increased from 8.6 min (IQR 5.0–24.8) to 19.3 min (IQR 6.0–53.8). Using the uncorrected times, an overall difference between study groups was detected (Kruskal–Wallis, *p* = 0.008), with post hoc analysis identifying a significant difference between IV 3 and IV 1 (Dunn’s test with Bonferroni correction, *p* = 0.005, r = 0.63).

No statistically significant association was observed between corrected time to first treatment order and patient triage category within any study group (Kruskal–Wallis: CONT: *p* = 0.22; IV 1: *p* = 0.44; IV 2: *p* = 0.74; IV 3: *p* = 0.22).

### 3.3. Indicated Treatments Addressed

The proportion of indicated treatments addressed differed across study groups (Table 1). In CONT, 56% of indicated treatments were addressed (22/39; 95% CI 41.0–70.7%), 8% in IV 1 (3/40; 95% CI 2.6–19.9%), 57% in IV 2 (28/49; 95% CI 43.3–70.0%), and 51% in IV 3 (23/45; 95% CI 37.0–65.0%). Pearson’s chi squared test showed a significant association between study group and receipt of indicated treatments (*p* < 0.001), with a moderate effect size (ω = 0.41).

Post hoc pairwise comparisons using Pearson’s chi squared tests with Bonferroni correction for six comparisons (adjusted α = 0.0083) were conducted. No significant differences were observed between CONT, IV 2, and IV 3. The 95% CIs for the corresponding between-group differences all included zero (Table A1). All comparisons involving IV 1 (EMT-only care) were significant (*p* < 0.001 in all comparisons), with medium-to-large effect sizes (ω = 0.47–0.53). Compared with IV 1, the proportion of indicated treatments addressed was higher by 48.9 percentage points in CONT (95% CI 29.1–64.0), 49.6 percentage points in IV 2 (95% CI 31.1–63.4), and 43.6 percentage points in IV 3 (95% CI 24.9–58.4).

### 3.4. Quality of Analgesia Management

#### 3.4.1. Percentage of Patients in Pain with Analgesia Addressed

**Figure 4:**
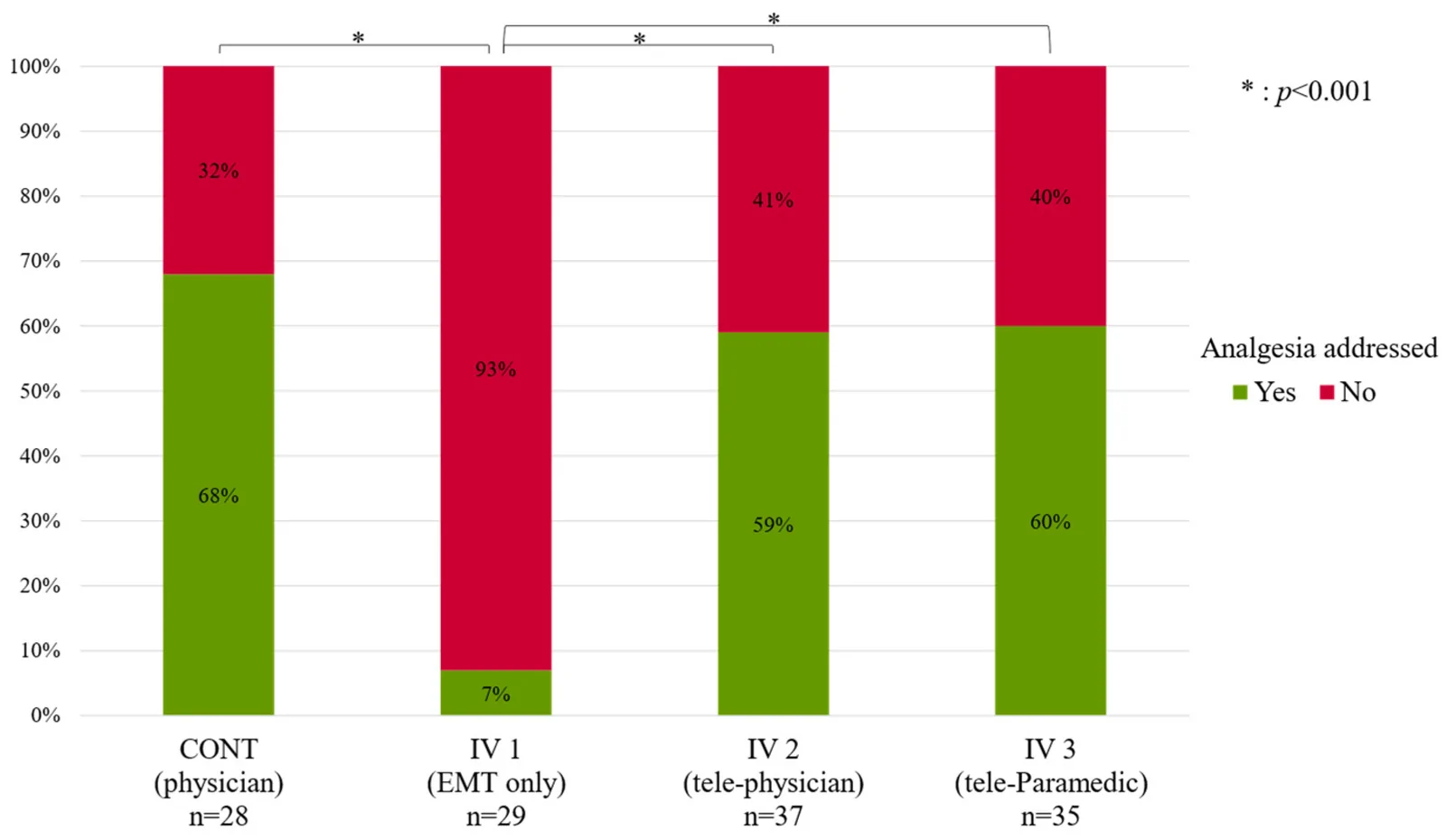
Percentage of patients in pain with analgesia addressed.

As shown in Figure 4, analgesia, indicated in 85% (129/151) of all patients, was addressed in 68% of patients in CONT (19/28; 95% CI 49.3–82.1%), compared with 59% (22/37; 95% CI 43.5–73.7%) of patients in IV 2 and in 60% in IV 3 (21/35; 95% CI 43.6–74.4%). In contrast, analgesia was addressed in only 7% of patients in IV 1 (2/29; 95% CI 1.9–22.0%), while it remained unaddressed in 93%.

There was a significant association between study group and analgesia addressed (Pearson’s chi squared test, *p* < 0.001, ω = 0.45). Post hoc pairwise comparisons using Pearson’s chi squared tests with Bonferroni correction for six comparisons (adjusted α = 0.0083) were conducted. No significant differences were observed between CONT, IV 2, and IV 3. The 95% CIs for the corresponding between-group differences all included zero (Table A2). However, all comparisons involving IV 1 (EMT-only care) were significant (*p* < 0.001 in all comparisons), with large effect sizes (ω = 0.54–0.63). Compared with IV 1, the proportion of patients with analgesia addressed was higher by 61.0 percentage points in CONT (95% CI 37.1–76.0), 52.6 percentage points in IV 2 (95% CI 30.6–67.6), and 53.1 percentage points in IV 3 (95% CI 30.8–68.4).

#### 3.4.2. Time to Analgesia Order

Time to analgesia order varied descriptively between groups but without statistical significance. Median times were 12.10 min (IQR 7.77–15.30) in CONT (*n* = 19), 8.24 min (IQR 4.81–16.58) in IV 2 (*n* = 22), and 21.75 min (IQR 9.23–53.24) in IV 3 (*n* = 17); data from IV 1 (*n* = 2) were not interpretable. A Kruskal–Wallis test revealed no significant differences between groups (*p* = 0.083).

No significant association was observed between time to analgesia order and patient triage category in three study groups (Kruskal–Wallis: CONT: *p* = 0.28; IV 2: *p* = 0.91; IV 3: *p* = 0.60; IV 1 was not tested due to small sample size).

#### 3.4.3. Influence of NRS Pain Level on Analgesia

**Figure 5:**
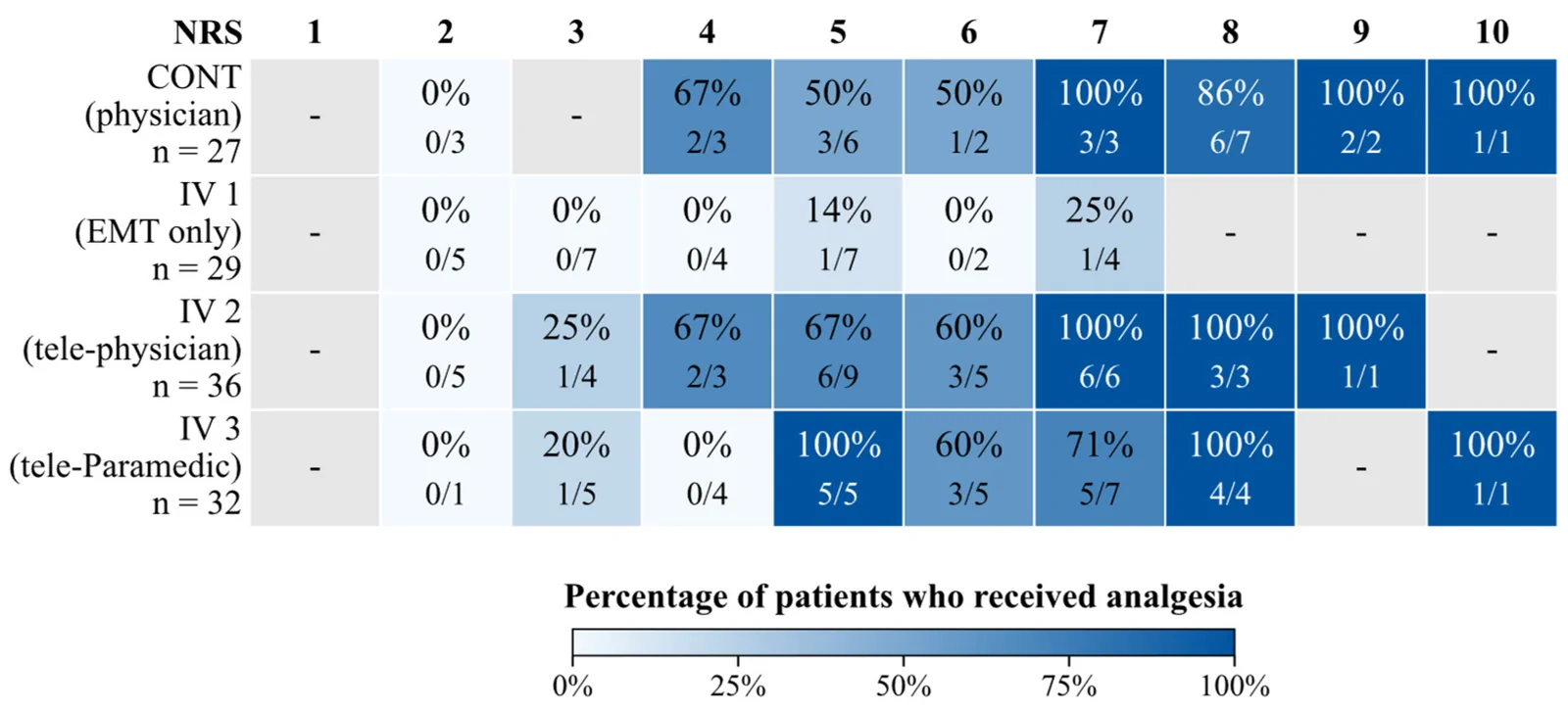
Analgesia rates across NRS pain levels by study group.

Consistent with the analgesia rates displayed in Figure 5, higher NRS values were associated with higher odds of analgesia being addressed in binary logistic regression in CONT (OR 2.06, 95% CI 1.16–3.66; *p* = 0.01; Nagelkerke’s R^2^ = 0.43), IV 2 (OR 2.72, 95% CI 1.44–5.17; *p* = 0.002; Nagelkerke’s R^2^ = 0.52), and IV 3 (OR 2.24, 95% CI 1.27–3.94; *p* = 0.006; Nagelkerke’s R^2^ = 0.41). In the EMT-only group, the association was not statistically significant (OR 2.30, 95% CI 0.72–7.34; *p* = 0.16; Nagelkerke’s R^2^ = 0.23).

The Box–Tidwell interaction term was not statistically significant in CONT (*p* = 0.671), IV 2 (*p* = 0.735), or IV 3 (*p* = 0.609), providing no evidence of violation of the linearity-in-the-logit assumption. This assumption was not assessed in IV 1 because analgesia was addressed in only two patients.

#### 3.4.4. Telemedicine Groups: Source of Analgesia Order

In both telemedicine groups (IV 2 and 3), most analgesia orders were placed via telemedicine. In IV 2 (tele-physician), 86% (19/22) of analgesia orders were issued through telemedicine and 14% (3/22) by the mobile medical response team, whereas in IV 3 (tele-Paramedic), 67% (14/21) were issued via telemedicine and 33% (7/21) by the mobile medical response team. Fisher’s exact test did not show a significant association between study groups and the source of analgesia orders (*p* = 0.16).

### 3.5. Patient Contacts

Across the four study groups, patient contact frequency varied. The highest contact rate was observed in IV 3 (19.60 contacts per hour), followed by IV 2 (13.23 contacts per hour). Lower contact frequencies were recorded in IV 1 (10.55 contacts per hour) and CONT (6.84 contacts per hour). Due to the absence of dispersion measures, no formal statistical comparison of group differences was made.

## 4. Discussion

This simulation study investigated whether telemedical support provided by either tele-physicians (IV 2) or tele-Paramedics (IV 3) can compensate for the absence of an on-site physician in disaster medicine. Both approaches were compared to a control group comprising a physician on site (CONT) and additionally to an EMT-only group (IV 1), representing a possible disaster scenario in which no personnel with advanced medical expertise are available because of staff shortages. The principal finding was that telemedical support provided by Paramedics with an advanced scope of practice showed similar observed performance to both tele-physician-supported and conventional physician-led care across several selected outcomes. Furthermore, both telemedicine groups had substantially higher rates of indicated treatments addressed and analgesia addressed. These findings suggest that access to advanced clinical expertise may be more important than the specific professional qualification of the tele-consultant; however, the absence of significant differences between tele-physician and tele-Paramedic support cannot be interpreted as evidence of equivalence.

Analysis of the distribution of triage categories revealed a significant difference only between the physician and EMT-only groups, whereas no significant differences were observed in comparisons involving either telemedicine group. These imbalances may have resulted from the operational allocation of patients and, in particular, from the request by one EMT-only tent not to receive patients triaged as red. Although no statistically significant differences in triage-category distribution were detected between the telemedicine groups and conventional physician-led care, some residual imbalance in patient acuity may still have been present and should be considered when interpreting the findings.

A major finding of this study is that no significant differences were observed between study groups in the time to first treatment order by the respective most highly qualified team members when periods of telemedicine unavailability were accounted for. Notably, this outcome reflects the contribution of the most highly qualified team members rather than the overall performance of the treatment team. Correcting for periods of telemedicine unavailability allowed the analysis to focus on the clinical performance of the telemedical care model rather than on limitations of the experimental technical system. In the sensitivity analysis using uncorrected elapsed times, however, an overall difference between study groups was detected, with significantly longer times in the tele-Paramedic group than in the EMT-only group. This finding indicates that technical unavailability contributed to the operational time to treatment order and highlights the distinction between clinical performance under functioning telemedical conditions and the elapsed time experienced during the exercise. The corrected findings are consistent with previous disaster telemedicine research. Müller et al. demonstrated that telemedicine can provide patient care comparable to conventional disaster medicine [7], while Boyle et al. similarly reported high acceptance of telemedicine for disaster response simulations [14]. A recent systematic review likewise identified telemedicine as a means of maintaining healthcare delivery during natural disasters while highlighting infrastructural and technological disruptions as recurring barriers to its implementation [15]. Overall, the present findings support the feasibility of telemedical consultation for initial treatment decision-making while also emphasizing that technical availability remains relevant to real-world operational performance.

No association was observed between triage category and time to first treatment order. This suggests that higher-acuity patients did not systematically receive earlier treatment orders within the study groups. In a disaster setting, where prioritization according to urgency is a central principle of care, this finding may indicate that factors other than triage category influenced the timing of initial treatment decisions. Another possible explanation is that in this simulation, study patients entered the treatment tents at sufficiently spaced intervals, potentially reducing the need for prioritization. However, the relatively small subgroup sizes limit conclusions regarding prioritization of individual triage categories.

The proportion of indicated treatments addressed differed significantly between study groups. The lowest proportion was observed in the EMT-only group (8%), whereas higher proportions were observed in the tele-physician (56%), tele-Paramedic (51%), and on-site physician (57%) groups. This finding supports the concept that telemedicine can extend higher-level medical expertise to treatment teams where such expertise is not physically available [16]. Previous simulation studies have demonstrated that relevant interventions can be delegated and guided telemedically without significant delays, even when performed by responders with comparatively limited medical qualifications [7,8]. Evidence from routine prehospital tele-EMS further indicates that remote physicians can support Paramedics through treatment decisions and delegated medication administration in potentially life-threatening emergencies [4]. The present findings extend this evidence by suggesting that telemedical support may not only enable individual interventions but may be associated with a higher proportion of indicated treatments addressed during a mass casualty scenario. Although rates of indicated treatments addressed remained incomplete even in the physician and telemedicine groups, these rates reflect a comparatively demanding subset of treatments, as only measures that would routinely require physician involvement were included. Nevertheless, access to advanced expertise alone did not ensure implementation of all indicated measures under disaster conditions.

Undertreatment of pain remains a persistent challenge in emergency and disaster medicine [17]. In the present study, analgesia was addressed in approximately two-thirds of patients with indication in the physician, tele-physician and tele-Paramedic groups, compared with only 7% in the EMT-only group. The very low rate of analgesia in the EMT-only group can be explained by the fact that EMTs in this study were legally not allowed to administer analgesia independently.

Time to analgesia order did not differ significantly between study groups, although the tele-Paramedic group showed descriptively longer and more variable times.

No statistically significant difference in time to analgesia order was detected between groups. However, the small sample sizes and wide variability, particularly in the tele-Paramedic group, preclude conclusions regarding similar treatment timing between care models. The absence of a significant association with triage category further indicates that analgesia ordering was not systematically faster in patients assigned to higher-acuity categories. Given the small sample sizes, particularly in the EMT-only group, and the wide variability observed in the tele-Paramedic group, these findings should be interpreted cautiously.

In all groups with access to advanced medical expertise but not in the EMT-only group, increasing pain severity significantly increased the likelihood of receiving analgesia. Taken together with the higher analgesia rates, this pattern suggests that access to advanced clinical expertise was associated not only with more frequent analgesia provision, but also with treatment decisions that reflected reported pain intensity.

Although overall analgesia rates were similar, the mobile medical response team was involved more often in analgesia provision in the tele-Paramedic group than in the tele-physician group (33% vs. 14%), but this difference did not reach statistical significance. This greater descriptive reliance on on-site support may have contributed to the overall analgesia rate observed in the tele-Paramedic group and should therefore be considered part of the performance of this care model. The present data do not allow firm conclusions regarding differences in escalation requirements. A separate analysis of the reasons for consulting the mobile medical response team is currently in progress. A notable finding was the high patient contact frequency observed in the telemedicine groups, particularly among tele-Paramedics. Previous work by Müller et al. reported that continuous teleconsultation increases patient contacts and may improve situational awareness [7].

In the present study, tele-consultants recorded more patient contacts per hour than the on-site physician. This may indicate that telemedicine facilitates frequent reassessment and ongoing oversight of multiple patients. However, patient contacts were defined differently between telemedicine and non-telemedicine groups: switching a remote conversation to another patient was counted as contact in the telemedicine groups, whereas physical movement between patients constituted contact in the non-telemedicine groups. These contacts may therefore differ in duration, clinical content, and intensity and should not be interpreted as directly equivalent. The observed pattern therefore warrants further investigation of whether telemedical workflows affect opportunities for reassessment and ongoing oversight.

The finding that tele-Paramedics showed similar observed performance to tele-physicians across several selected outcomes represents the most important contribution of this study. Previous disaster telemedicine research has largely focused on physician-to-provider teleconsultation models [4,7,8,18,19], and direct comparison with Paramedic tele-consultants has not been reported. The present findings suggest that experienced Paramedics may be able to provide remote support for selected disaster medicine tasks, particularly when working with structured treatment algorithms and established disaster medicine guidelines. This is especially relevant given projected physician shortages and the substantial prehospital expertise of Paramedics. Expanding tele-consultant roles to this professional group could therefore increase the pool of available remote expertise and strengthen surge capacity during large-scale incidents. However, these preliminary findings require confirmation in adequately powered studies, preferably using multicenter or cluster-based designs.

Several limitations should be considered when interpreting these findings. This was a simulation study and therefore cannot fully reproduce the operational complexity and psychological stress of real disasters. Simulated patients cannot completely replicate the behavior, appearance, and emotional responses of real casualties. The study focused on thermal and traumatic injuries, which is reflected in the chosen outcome parameters. Therefore, the findings cannot be generalized to all relevant disaster scenarios and civil protection interventions.

Furthermore, the study was conducted within the German civil protection system and may not be directly transferable to healthcare systems with different organizational structures or scopes of practice for Paramedics. Participant and patient allocation introduced additional potential sources of confounding. Participant experience varied. Some EMTs were actively working in emergency medical services and therefore had greater practical experience than others. No randomization was performed to account for these differences because participant allocation was determined by the training exercise. In addition, one tele-physician was familiar with both the study’s telemedicine system and an established tele-emergency physician system, which may have provided an advantage. However, increasing adoption of telemedicine in emergency medical services is likely to make such prior experiences more common. Patient allocation to treatment tents was likewise not randomized, and the resulting imbalance in triage categories introduces the possibility of residual confounding by case mix, which could not be fully accounted for in the present analyses.

An important limitation is that study condition was assigned at the treatment-tent level, whereas statistical analyses were conducted at the patient level. Patients treated within the same tent were managed by the same team and may therefore not represent fully independent observations. With only seven treatment tents, including a single control tent, reliable adjustment for intracluster correlation was not feasible. Consequently, patient-level analyses may overestimate the precision of between-group comparisons, and effects attributable to the care model cannot be fully separated from tent- or team-specific effects. This also introduces the possibility of pseudoreplication, as multiple patient-level observations were analyzed within a comparatively small number of tent-level experimental units.

The primary outcome focused exclusively on treatment orders issued by the most highly qualified team member and therefore does not reflect the overall performance of the respective treatment team. Restricting this analysis to patients with at least one documented treatment order by the most highly qualified team member may also have introduced selection bias if the likelihood of such an order differed between study groups.

Rates of indicated treatments addressed may have been influenced by unequal access to information. The guidelines used to determine treatment indication had been introduced only six months before the study, and participants were provided an abbreviated version during the study that was not routinely available. Tele-consultants may have benefited from easier access to additional information through digital technology than personnel working in the non-telemedicine tents.

The sample size was limited for several analyses, and no formal a priori sample-size or power calculation was performed. The study was also not designed as an equivalence or non-inferiority trial, and no predefined equivalence or non-inferiority margins were specified. Accordingly, the absence of statistically significant differences cannot be interpreted as evidence of equivalence between care models. In addition, the number of statistical comparisons increased the risk of type I error.

Finally, limitations related to data collection and technical performance may have affected several outcome measures. Treatment orders and patient contacts had to be reconstructed from video recordings and observer notes, and exact time points could not always be determined. Furthermore, patient contacts were operationally defined differently in telemedicine and non-telemedicine groups and therefore do not represent directly equivalent measures of provider–patient interaction. In addition, some data were lost because of camera failures, incomplete screen recordings, and intermittent Wi-Fi connectivity. These technical issues likely influenced several outcome measures but also reflect challenges that may occur during real disaster responses.

Future research should define which disaster medicine tasks can be performed by tele-Paramedics, which can safely be delegated through telemedicine, and when physician involvement or on-site presence remains essential. Larger simulation studies and real-world deployments should evaluate patient-centered outcomes, resource utilization and cost-effectiveness. Furthermore, hybrid consultation models in which tele-Paramedics provide first-line support while tele-physicians remain available for escalation of complex cases should be investigated. Future evaluations of hybrid telemedical models should also consider provider acceptability, communication quality, and the influence of familiarity and trust between on-site and remote professionals.

## 5. Conclusions

The present findings suggest that telemedical support may help address shortages of highly qualified medical personnel in disaster medicine. Across several selected outcomes, tele-physician and tele-Paramedic support showed similar observed performance to conventional physician-led care, while EMT-only care showed substantially lower rates of indicated treatments addressed and analgesia provision. This suggests that telemedical access to advanced clinical expertise can provide meaningful added value when such expertise is otherwise unavailable on site. Within this context, experienced Paramedics may represent a valuable and underutilized resource for telemedical disaster response, potentially expanding access to advanced clinical expertise during major incidents.

Further research should define the appropriate scope of tele-Paramedic practice and escalation criteria, and evaluate effectiveness, scalability, and operational requirements of hybrid tele-Paramedic/tele-physician models in larger simulations and real-world disaster settings.

## Figures and Tables

**Table 1:** Rates of most frequently indicated treatments addressed.

	CONT (Physician)	IV 1 (EMT Only)	IV 2 (Tele-Physician)	IV 3 (Tele-Paramedic)
Total	22/39 (56%)	3/40 (8%)	28/49 (57%)	23/45 (51%)
Analgesia	19/28 (68%)	2/29 (7%)	22/37 (59%)	21/35 (60%)
Volume substitution	2/3 (67%)	1/2 (50%)	4/5 (80%)	2/3 (67%)
Fracture reduction	0/1 (0%)	0/5 (0%)	2/3 (67%)	0/1 (0%)
Antibiotics	0/1 (0%)	0/2 (0%)	0/2 (0%)	0/1 (0%)
Oxygen-therapy	-	-	0/1 (0%)	0/2 (0%)
Acetylsalicylic acid	0/1 (0%)	0/1 (0%)	-	-
Lowering blood pressure	1/1 (100%)	0/1 (0%)	-	-
Pelvic sling	0/1 (0%)	-	-	0/1 (0%)
Thoracic decompression	0/1 (0%)	-	-	0/1 (0%)
Tourniquet	0/1 (0%)	-	-	0/1 (0%)
Tranexamic acid	0/1 (0%)	-	0/1 (0%)	-

## Data Availability

The original data presented in the study are openly available on FigShare at DOI: 10.6084/m9.figshare.33265563.

## Abbreviations

The following abbreviations are used in this manuscript:

CI	Confidence Interval
CONT	Control group (physician on site)
EMR	Emergency Medical Responder
EMS	Emergency Medical Services
EMT	Emergency Medical Technician
IQR	Interquartile Range
IV 1	Intervention Group 1 (EMTs or lower)
IV 2	Intervention Group 2 (tele-physician)
IV 3	Intervention Group 3 (tele-Paramedic)
NHTSA	National Highway Traffic Safety Administration
NRS	Numeric Rating Scale
OR	Odds Ratio
PIVC	Peripheral Intravenous Catheter

## Appendix A

### Appendix A.1

**Table A1:** Pairwise differences in the proportion of indicated treatments addressed.

Comparison	Difference in Proportion, Percentage Points	95% CI
CONT–IV 1	+48.9	29.1–64.0
IV 2–IV 1	+49.6	31.1–63.4
IV 3–IV 1	+43.6	24.9–58.4
CONT–IV 2	−0.7	−20.8–19.2
CONT–IV 3	+5.3	−15.5–25.4
IV 2–IV 3	+6.0	−13.6–25.1

### Appendix A.2

**Table A2:** Pairwise differences in the proportion of patients with analgesia addressed.

Comparison	Difference in Proportion, Percentage Points	95% CI
CONT–IV 1	+61.0	37.1–76.0
IV 2–IV 1	+52.6	30.6–67.6
IV 3–IV 1	+53.1	30.8–68.4
CONT–IV 2	+8.4	−14.9–29.8
CONT–IV 3	+7.9	−15.6–29.6
IV 2–IV 3	−0.5	−22.1–21.2
